# Spherical Equivalent Percentile Curves in a Portuguese School-Aged Population

**DOI:** 10.3390/jcm14207262

**Published:** 2025-10-14

**Authors:** María Ibeth Peñaloza-Barbosa, Clara Martinez-Perez, Cristina Andreu-Vázquez, Miguel Ángel Sánchez-Tena, Cristina Alvarez-Peregrina

**Affiliations:** 1 School for Doctoral Studies and Research, Universidad Europea de Madrid, 28670 Madrid, Spain; mariaibeth2008@gmail.com; 2 Instituto Superior de Educação e Ciências de Lisboa (ISEC Lisboa), Alameda das Linhas de Torres, 179, 1750-142 Lisboa, Portugal; 3 Department of Veterinary Medicine, Faculty of Biomedical and Health Sciences, Universidad Europea de Madrid, 28670 Madrid, Spain; cristina.andreu@universidadeuropea.es; 4 Department of Optometry and Vision, Faculty of Optics and Optometry, Universidad Complutense de Madrid, 28037 Madrid, Spain; cristina_alvarez@ucm.es

**Keywords:** percentiles, myopia, hyperopia, refractive errors, children, adolescents

## Abstract

**Bacground/Objectives**: This study aimed to develop age- and sex-specific spherical equivalent (SE) percentile curves and estimate the prevalence of refractive errors (REs) in Portuguese schoolchildren aged 6–17 years. **Methods**: A cross-sectional study was conducted in three schools in Lisbon, including 2205 children (mean age = 9.3 ± 2.6 years; 49.3% boys). Vision was assessed using non-cycloplegic static retinoscopy (chosen due to feasibility in school settings) and visual acuity tests. SE percentile curves (P5–P95) were generated by age and sex. SE values and RE distribution (hyperopia, emmetropia, and myopia) were compared across four age groups (6–8, 9–11, 12–14, and 15–17 years). **Results**: SE values decreased significantly with age (*p* < 0.001). Median SE ranged from +0.50 D (6–8 years) to 0.00 D (15–17 years), with no sex differences. Hyperopia predominated in younger children (60.6% at 6–8 years), whereas myopia increased in older ages (32.2% at 15–17 years). **Conclusions**: This study presents the first SE percentile curves for Portuguese schoolchildren, providing a practical, age-specific reference for vision screening. The progressive shift from hyperopia to myopia highlights the importance of early detection and monitoring to prevent visual impairment.

## 1. Introduction

Emmetropization is a visually guided developmental process in which the axial growth of the eye is coordinated with changes in the refractive power of its optical components, primarily the cornea and crystalline lens, to achieve and maintain a state of minimal refractive error (RE) without relying on accommodation for distant vision [1]. This mechanism, which minimises RE, is influenced by various environmental (e.g., cultural, ambient illumination, light intensity) and lifestyle factors (e.g., outdoor activity time, nutrition, genetics) [2,3].

Refractive development evolves with age, starting with moderate hyperopia at birth, which typically decreases by the age of six [2,4]. Early changes include adjustments to corneal curvature and a reduction in the crystalline lens dioptric power. After the age of six, other RE such as myopia may commonly develop [4]. Therefore, early detection is crucial to prevent amblyopia and strabismus [2,5,6]. In Europe, myopia increases with age, particularly during adolescence, while hyperopia decreases, and is more common in early school years [7]. From adolescence, myopia is the most common RE, and its prevalence has increased in recent decades. It is estimated that myopia will affect almost half of the world’s population by 2050, with a higher prevalence in East Asia, followed by European and African populations [8]. Similar trends are expected in Portugal, but the scarcity and data heterogeneity on RE progression in the Portuguese school population do not allow for clear age-based segregation [9].

In paediatrics, percentile curves are an excellent tool to assess the age distribution of a variable compared to a reference population [10,11]. Their application to refractive status is particularly relevant during childhood, as RE is a global health problem [8]. Recent studies have highlighted their usefulness as a reliable diagnostic measure to detect these errors in children [11,12]. Furthermore, curves characteristics vary according to the population studied, so it is essential to consider the RE prevalence in each context, such as in China, where myopia affects 80% of the population [13,14].

In contrast to other European countries, the use of percentile curves as a standard tool in clinical practice in Portugal has not been achieved yet. Nonetheless, recent research indicates that the prevalence of RE in the Portuguese population rises with age (31.9%), highlighting the urgent need for public health initiatives focused on early detection and correction [9].

Most studies on RE in childhood populations have been conducted in Asian populations [4,15,16,17,18]. In Europe, the availability of such studies is limited [12,19,20,21,22] and, to date, scarce research in this area has been developed in Portugal. Given the proven effectiveness of percentile curves in identifying refractive status at early ages, the present study aims to develop SE percentile curves for a Portuguese school-aged population (6 to 17 years old). As a secondary objective, the study will also assess the prevalence of refractive errors within this population.

While several studies in European countries have described refractive development in children [12,19,20,22], data from Portugal remain scarce and fragmented, limiting the ability to establish population-specific references [9,21]. The Portuguese school-aged population has not yet been characterized through age- and sex-specific percentile curves, despite evidence suggesting an increasing prevalence of refractive errors with age [9]. Addressing this gap is crucial to support early detection strategies and guide public health policies. Therefore, this study aims to provide the first normative reference for visual development in Portuguese children, contributing new epidemiological data to the European context [11,12,22].

## 2. Materials and Methods

### 2.1. Study Design

This was an observational, descriptive, and cross-sectional study. The research adhered to the principles of the Declaration of Helsinki and was approved by the Ethics Committee of the Higher Institute of Education and Sciences (ISEC) in Lisbon (Portugal) (CE/2022/03/01). Parents or legal guardians of all participants signed an informed consent document.

### 2.2. Study Population

Between July and September 2021, 2022, and 2023, invitation letters were sent to seven schools in Lisbon. Three schools agreed to participate: *Agrupamento de Escolas Póvoa De Santa Iria* (Vila Franca de Xira, Lisbon, Portugal), *Agrupamento de Escolas de Alcabideche* (Cascais, Lisbon, Portugal), and *Colégio Campo de Flores* (Almada, Lisbon, Portugal). Children aged 6 to 17 years whose parents provided informed consent were included. Children with any ocular and/or systemic disease reported by their parents were not included in the study. Data collection took place during the 2021–2022, 2022–2023, and 2023–2024 academic years. Each child was assessed only once, with no repeated evaluations across the years. The same age range (6 to 17 years) was included in each academic year, depending on the age distribution at each participating school.

### 2.3. Procedure

The procedures carried out were non-cycloplegic static retinoscopy to assess refractive status and visual acuity measurements. The non-cycloplegic retinoscopy was performed using the Directional E and Snellen (mixed) optotypes, placed at eye level at three metres. Children were instructed to fixate on the optotype while the examiner, positioned at 60 cm, evaluated the retinal reflex, controlled accommodation, and neutralised the shadows using the appropriate lenses: negative for myopia, positive for hyperopia, and cylindrical for astigmatism [23]. All examinations were performed by experienced optometrists. The following variables were recorded for each participant: cylinder, sphere, and spherical equivalent (SE), calculated as SE = Sphere + (Cylinder/2).

Optometrists performed the screenings at the participating schools. Each school prepared an appropriate and well-equipped room for testing, where subjects were organised by age and school grade. The designated room had two separate areas: one to accommodate the group and another for individual testing. Although children attended in groups, all tests were conducted individually to prevent interaction and avoid potential bias in data collection.

Refractive error classification followed the definitions proposed by the International Myopia Institute (IMI) 2023 Digest [24] and was expressed in negative-cylinder notation:

- Myopia: SE ≤ −0.50 D;
- Hyperopia: SE ≥ +0.50 D;
- Emmetropia: SE between −0.50 D and +0.50 D;
- Astigmatism: Cylinder < 0.00 D (negative-cylinder notation).

### 2.4. Statistical Analysis

Continuous variables are expressed as means ± standard deviations (SD), as well as medians and interquartile ranges [P25, P75], and categorical variables are reported as absolute (n) and relative (%) frequencies. Percentile values (P5, P10, P25, P50, P75, P90, and P95) of SE were calculated for the right eye across the entire sample and stratified by age and sex. In order to evaluate age-related trends in SE, individual age was classified into four categories: 6–8, 9–11, 12–14, and 15–17 years [14,17,20,25,26,27,28]. Differences in SE values across age categories were assessed using the Kruskal–Wallis test, followed by Bonferroni-corrected pairwise comparisons. Differences in SE between sexes were assessed using the Mann–Whitney U test for the entire sample, and for each age category. The distribution of RE types (hyperopia, emmetropia, and myopia), as well as the presence of astigmatism, across age categories was compared using chi-square tests. Additionally, differences in SE values were assessed across age categories separately within each RE group (hyperopic, emmetropic, and myopic) using the Kruskal–Wallis test to assess age-related differences within each group. All analyses were performed using STATA v.17 (StataCorp LLC, College Station, TX, USA), and a significance level of 5% was used.

## 3. Results

A total of 2228 children aged 6 to 17 years underwent visual examinations. Twenty-three children (1.03%) were excluded due to missing data on SE measurements in both eyes. The final sample included 2205 children (49.3% male; mean age: 9.33 ± 2.56 years). Table 1 presents the age distribution of the entire sample, stratified by sex.

Table 2 presents the mean, standard deviation, and the 25th, 50th, and 75th percentiles of sphere, cylinder, and SE values across the four age categories (6–8, 9–11, 12–14, and 15–17 years). Detailed description of sphere, cylinder, and SE for each age, for the overall sample, and stratified by sex, is available in Supplementary Table S1.

**Figure 1:**
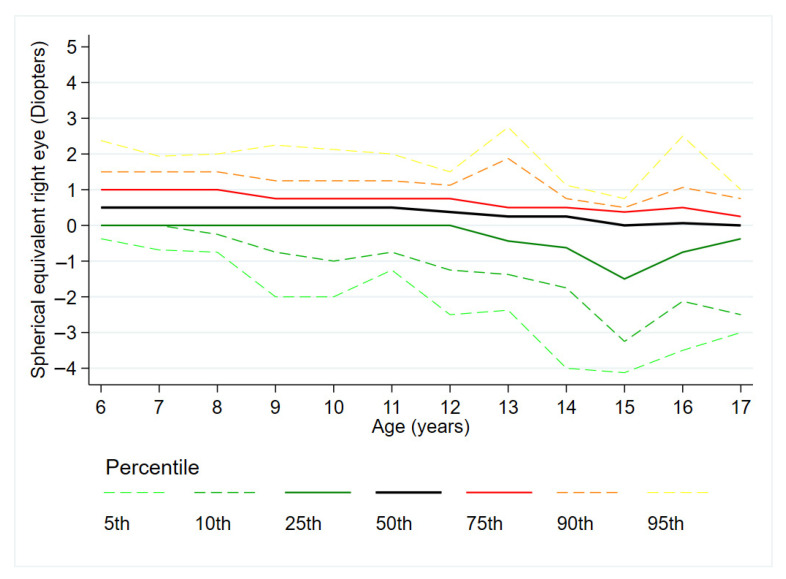
Percentile curves of spherical equivalent (right eye) from ages 6 to 17 years.

**Figure 2:**
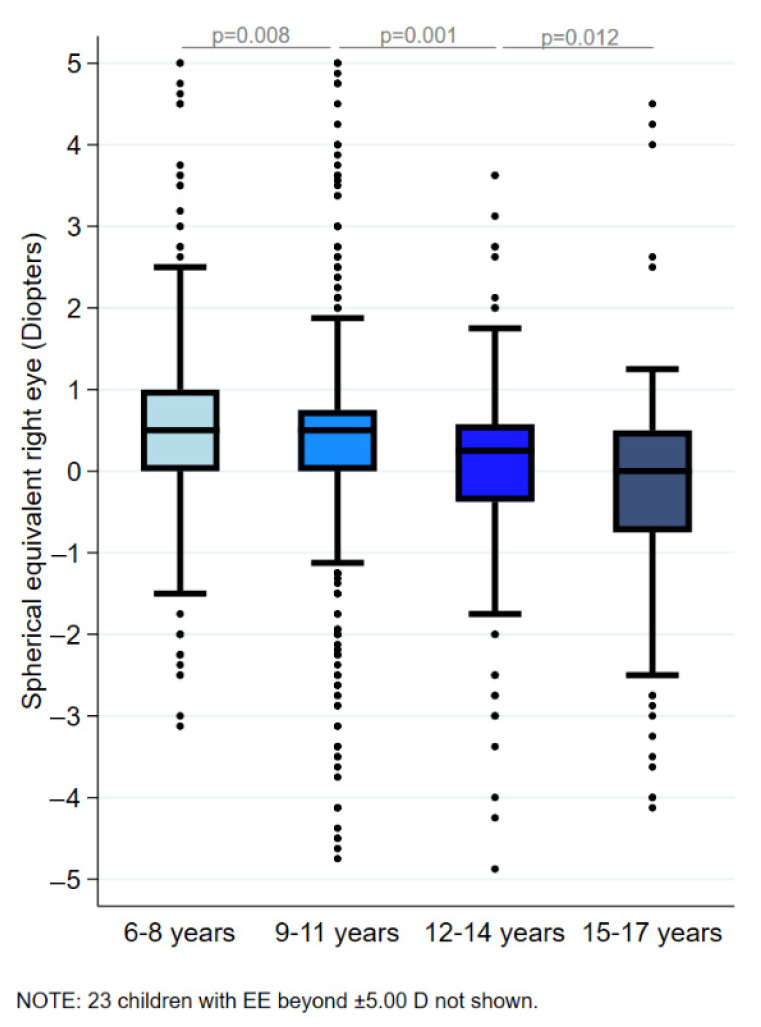
Box plot of spherical equivalent (right eye) across age categories. The line splitting the box in two represents the median value. The box represents the interquartile range of samples. The upper edge of the box represents the upper quartile (Q3). The lower edge of the box shows the lower quartile (Q1). The values at which the whiskers stop are the values of the upper and lower values of the data. The single points on the diagram show the outliers. The measurements of SE were taken in the right eye. n = 2205.

Figure 1 displays the percentile curves (5th, 10th, 25th, 50th, 75th, and 95th) of SE across ages 6 to 17 years. SE values differed significantly across age ranges (Kruskal–Wallis test *p* < 0.001). Bonferroni-corrected pairwise comparisons revealed a progressive and statistically significant decrease in SE with increasing age categories: children aged 6–8 years had higher SE values than those aged 9–11 years (*p* = 0.008); children aged 9–11 years had higher values than those aged 12–14 years (*p* = 0.001); and those aged 12–14 years had higher values than children aged 15–17 years (*p* = 0.012; Figure 2). No significant differences in SE were observed between boys and girls, either in the overall sample (Mann–Whitney U test *p* = 0.423), or within each age range (Mann–Whitney U tests *p* = 0.929, 0.453, 0.509, and 0.228 for the 6–8, 9–11, 12–14, and 15–17 year age categories, respectively).

**Figure 3:**
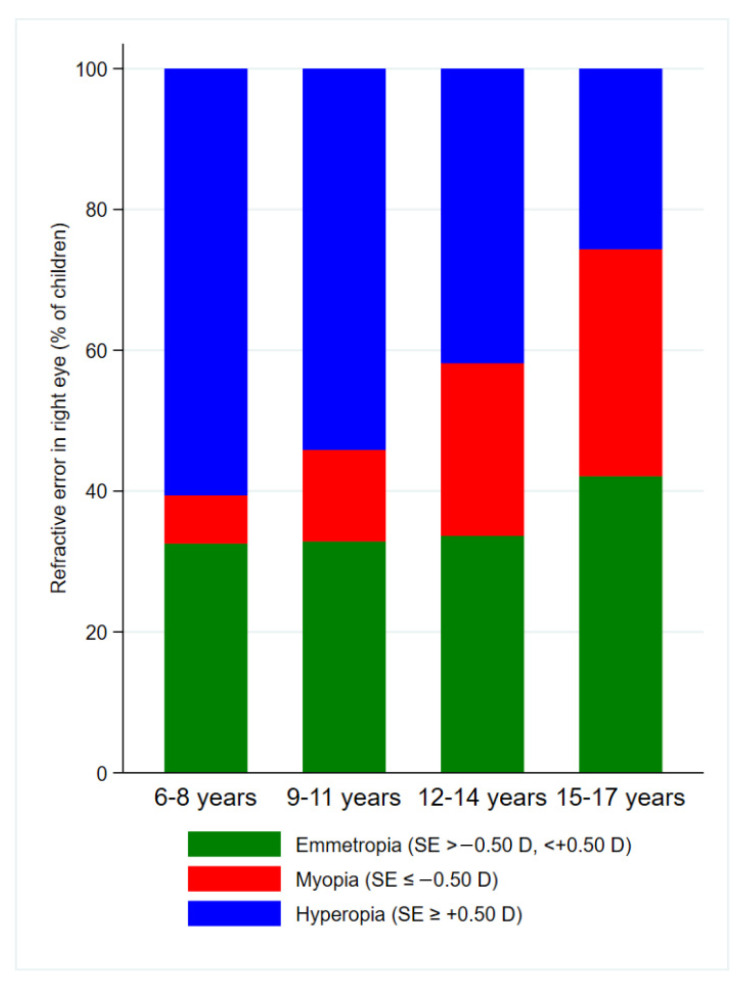
Distribution of refractive error types (right eye) across age categories.

Overall, 54.1% of the children evaluated were classified as hyperopic, 33.3% as emmetropic, and 12.6% as myopic. The distribution of RE types differed significantly among the four age categories (chi-square test, *p* < 0.001; Figure 3). While the proportion of children with myopia increased progressively with age, the prevalence of hyperopia decreased. Specifically, hyperopia was the most common condition in younger children (60.6% in the 6–8-year-old group), but its prevalence declined to 25.6% in the 15–17-year-old group. In contrast, the prevalence of myopia rose from 6.8% in the youngest category to 32.2% in the oldest category.

**Figure 4:**
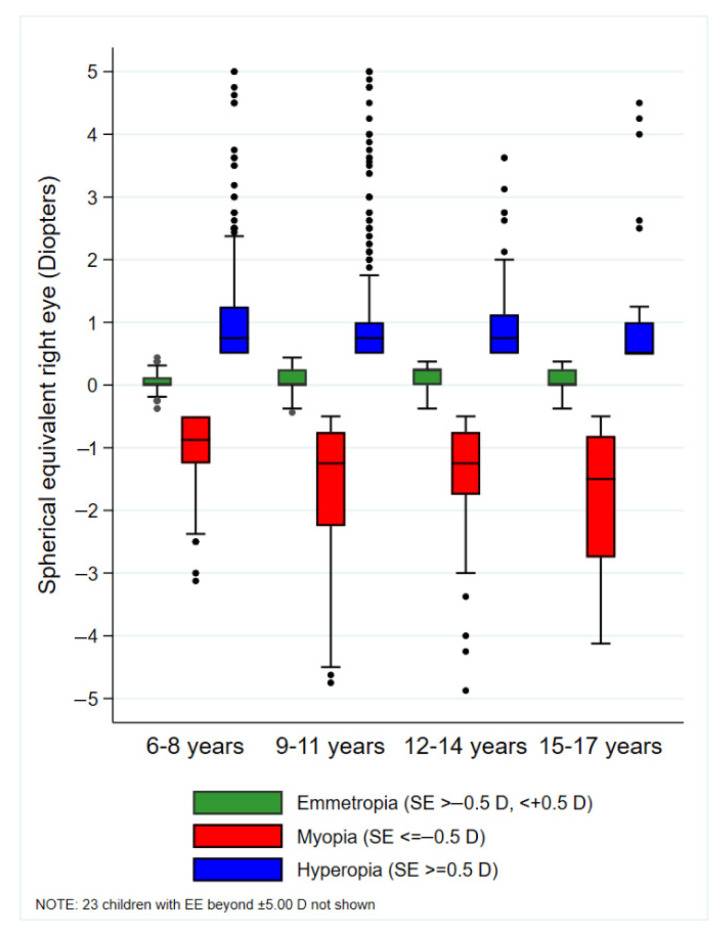
Box plot of spherical equivalent (right eye) by type of refractive error across age categories. The line splitting the box in two represents the median value. The box represents the interquartile range of samples. The upper edge of the box represents the upper quartile (Q3). The lower edge of the box shows the lower quartile (Q1). The values at which the whiskers stop are the values of the upper and lower values of the data. The single points on the diagram show the outliers. The measurements of SE were taken in the right eye. n = 2205.

SE in the right eye did not differ significantly across age categories in emmetropic (Kruskal–Wallis *p* = 0.200) or hyperopic children (Kruskal–Wallis *p* = 0.789). In contrast, in myopic children, SE values became progressively more negative with increasing age categories (Kruskal–Wallis *p* = 0.021; Figure 4).

## 4. Discussion

RE are common in the general population [5], and the uncorrected RE is the most important factor influencing visual impairment in children [29,30,31,32,33]. Epidemiological studies indicate a current trend of increasing myopia in childhood [4]. In this context, the present study provides a valuable contribution to the understanding of RE development in childhood, being the first to establish SE percentile curves for a Portuguese school-aged population. Our results indicate a progressive and significant decrease in SE with increasing age population. Our findings confirm that hyperopia is the most prevalent RE in younger children, while myopia becomes increasingly common during adolescence. These trends are consistent with previous studies conducted in both European and Asian populations [21,22,34,35].

Although cycloplegic refraction is considered the gold standard for assessing RE in children due to its ability to temporarily suspend accommodation, its use is limited in routine clinical practice across many European countries, including Portugal [36]. Legal and professional restrictions, along with practical concerns—such as potential side effects and the logistical challenge of applying cycloplegia to large paediatric populations—make its widespread use unfeasible [37]. Therefore, developing percentile curves based on non-cycloplegic measurements is both necessary and clinically relevant. These curves offer a practical tool for routine screenings, allowing clinicians to identify children whose RE deviates from age norms. In this context, a study carried out in German children reported a rapid decline in SE values towards myopia in the 3rd percentile curve (SE: −0.25 to −6.15D) [11]. Similarly, our data show a slightly lower but still clinically relevant shift in SE values at the lower percentiles, such as the 5th and 10th (SE: −0.38 to −4.13D; 0.00 to −3.25D, respectively). These findings underscore the high sensitivity of these lower percentiles for detecting children at elevated risk of developing high myopia. Nonetheless, in such cases, cycloplegic refraction can be recommended to confirm the diagnosis and guide appropriate intervention [38,39]. Thus, this methodological variability makes it difficult to compare our results with those of previous research, based on cycloplegic retinoscopy.

Retinoscopic spherical measurement is influenced by cycloplegia. SE values measured without cycloplegia can lead to an overestimation of myopia, as accommodation remains active during retinoscopy. In cases of latent hyperopia, the accommodative system may mask the RE. As a result, without cycloplegia, the refraction may underestimate the degree of hyperopia or, in some instances, even present as a false myopia. Nevertheless, as carried out in our study, using loose trial lenses during retinoscopy, as well as the observation of an optotype figure, it is possible to monitor the child’s eyes and fixation effectively, controlling the child’s accommodation at a distance in static retinoscopy, without the use of cycloplegic drugs [39,40]. Previous studies support the use of non-cycloplegic retinoscopy in young patients, such as Mohindra retinoscopy, with the exception of patients with high hyperopic refractions [41,42,43]. In general, cycloplegic retinoscopy is crucial for accurately detecting significant refractive errors in cases of strabismus, amblyopia, or anisometropia [38]. This, therefore, highlights the fact that non-cycloplegic retinoscopy can be used successfully as a screening tool for RE status, as it was conducted in our study.

The progressive shift from hyperopia to myopia with age observed in our sample aligns with the natural course of refractive development. Our study population is characterized by a 75th SE percentile curve ranging between +0.75 and +1.00 D between 6 and 12 years, becoming emmetropic from the age of 13 (SE: +0.25 to +0.50 D). In addition, according to a study developed in Germany, the 50th SE percentile curve presents positive values between 0.00 and +0.50 D, indicating an emmetropic median population [12]. However, other studies conducted among Spanish and Asian children, as well as a previous study carried out in Germany, showed a more myopic environment based on 50th and 75th SE percentiles curves, respectively [11,12,22]. In the Spanish study, the median of the 3–12-year-old children population showed negative SE values, typical of a myopic population (SE ≤ 0.50D) [22]. By contrast, the study performed in Chinese children reported a population that started to become myopic at 13 years, according to the 50th SE percentile curve [11]. These differences may be due to differences in population characteristics, environmental influences, and genetic factors [25,44,45]. It is known that myopia is more prevalent in Asian populations than in European ones, due to their greater axial length and corneal curvature [11,17,18]. These differences highlight the need to develop percentile curves that are specific to ethnicity and population [11,22]. Apart from the aforementioned factor, the variations observed in comparison to European populations could be partly related to the methodology used to measure RE. While our study employed a static non-cycloplegic retinoscopy, the other studies used autorefractometry with or without cycloplegia, which can significantly affect the classification of RE [11,12,22,39]. This indicates the necessity of a standardised methodology that allows for an accurate assessment of refractive status in childhood while conforming to local health regulations. Such alignment would also enhance the quality and comparability of global research, enabling more meaningful analyses across different populations.

Several studies agree that hyperopia is the most frequent RE in children aged 6 to 9 years. Studies in India, Jordan, and Polynesia report hyperopia rates between 60% and 65%, with SE ranging from +0.07 to +0.26 D [46,47,48]. In our study population, hyperopia is the most prevalent RE at younger ages (up to 12 years) and myopia, along with emmetropia, at older ages (between 13 and 16 years). Similarly, a study conducted in a population of Malaysian children aged 7–12 years is predominantly hyperopic (SE: +0.27 to +0.55 D) [26]. Additionally, Australian, Chinese, Brazilian, European, and Irish children aged 6 to 14 are predominantly hyperopic, although the dioptric values are higher than those of our Portuguese study population (SE: +0.23 to +9.38D) [19,20,27,49,50]. Hyperopia prevalence in Portuguese children aged 5 years is 14.2%, much lower than that of our study population at the same age (57%). Caution is advised when comparing our results with previous Portuguese studies, as differing diagnostic criteria—such as higher SE thresholds for defining hyperopia—limit direct comparability with results [21].

Some studies reveal a relation between refractive status prevalence and gender, mainly due to the overlapping of environmental, genetic, and physiological factors [11,12,28,44]. However, our investigation shows no difference in SE between boys and girls, regardless of the type of RE. Hyperopia was the most common condition in younger children (60.6% in the 6–8-year-old group), although its prevalence declined to 25.6% in the 15–17-year-old group. According to this, some studies suggest that RE prevalence depends more on age than on gender [1,34]. Therefore, further studies are needed to draw more robust conclusions.

RE severity is typically assessed using the SE values. Previous research has shown that percentile curves are highly effective in offering age-adjusted estimates of RE severity in school-aged children [11,12,22,51]. Nonetheless, key challenges remain: establishing clear, age-specific thresholds to define severe RE, as well as standardising the methodology and bringing it in line with local health regulations. This is crucial for detecting children at increased risk of progressing to high myopia, as early identification can help prevent long-term ocular complications.

One limitation of this study is the variability in refraction assessment methods reported in the literature, with some studies evaluating refraction under cycloplegia and others without, making direct comparisons challenging. Another limitation is the uneven distribution of subjects across age groups, which may affect the generalisability of the percentile curves and highlights the need for balanced representation in future studies. Additionally, various anatomical, physiological, and optical factors—such as retinal eccentricity, refractive error, pupil size, luminance, and contrast—may influence visual acuity measurements [52]. Although these factors were not addressed in our study, they should be considered in future research. Further studies should also investigate the relationship between refractive error prevalence and the time spent in outdoor versus near-vision activities.

A further limitation of this cross-sectional study is the absence of comprehensive ocular biometry, such as axial length measurements. This anatomical parameter is highly relevant for understanding refractive development, as known differences in myopia prevalence between Asian and European populations are partly attributed to greater axial length in Asian populations [3,10,15,16,17,18]. Despite this methodological omission, the SE percentile curves reported here maintain strong clinical utility and contextual relevance. They represent the first SE percentile reference for Portuguese schoolchildren, providing a practical, age-specific tool for vision screening and facilitating early identification of children at risk of refractive anomalies.

Despite these limitations, this approach can be considered reliable, as experienced optometrists performed the examinations and children were instructed to fixate on a distant target with the fellow eye. Moreover, non-cycloplegic static retinoscopy was selected for its feasibility, safety, and practicality in large school-based screenings, where the use of cycloplegia is often restricted. However, the cross-sectional nature of the study does not allow for longitudinal assessment of refractive development. Nevertheless, this study provides the first SE percentile curves for a Portuguese school-aged population, offering valuable data for early detection of refractive errors. These curves are highly relevant for establishing the relationship between age, sex, and refractive error prevalence during childhood, facilitating screening by health professionals and supporting preventive strategies. In the long term, this investigation may help inform public health policies by providing a baseline for exploring the factors that influence the visual health of Portuguese children.

## 5. Conclusions

This study presents, for the first time, SE percentile curves for a Portuguese school-aged population, providing a valuable reference for clinicians and public health professionals to identify children whose refractive development deviates from age-expected norms, enabling early detection and intervention.

Our findings show a progressive decrease in SE values with increasing age, consistent with the natural shift from hyperopia in early childhood to myopia during adolescence, and there were no differences between genders. While hyperopia was the most prevalent refractive error among younger children, myopia became increasingly common in older age groups.

These results highlight the importance of age-specific percentile curves as a practical screening tool in school settings and support their integration into vision monitoring programs to improve early diagnosis and management of refractive errors in children.

## Figures and Tables

**Table 1:** Age and sex distribution of the study sample.

	Total (n = 2205)	Boys (n = 1087; 49.3%)	Girls (n = 1118; 50.7%)
Age (mean ± SD)	9.33 ± 2.56	9.36 ± 2.50	9.30 ± 2.62
Age category (n, (%))			
6–8 years	921 (41.77)	442 (40.66)	479 (42.84)
9–11 years	955 (43.31)	479 (44.07)	476 (42.58)
12–14 years	208 (9.43)	113 (10.40)	95 (8.50)
15–17 years	121 (5.49)	53 (4.88)	68 (6.08)

SD, standard deviation.

**Table 2:** Sphere, cylinder (negative-cylinder notation), and spherical equivalent of the right eye (dioptres) in the study population.

	Mean	SD	p25	p50	p75
Sphere (D)					
6–8 years	0.72	1.15	0.00	0.50	1.00
9–11 years	0.70	1.50	0.25	0.50	1.00
12–14 years	0.28	1.31	0.00	0.50	0.75
15–17 years	−0.18	1.75	−0.50	0.00	0.50
Cylinder (D)					
6–8 years	−0.30	0.77	−0.25	0.00	0.00
9–11 years	−0.48	0.79	−0.50	−0.25	0.00
12–14 years	−0.48	0.75	−0.50	−0.25	0.00
15–17 years	−0.45	0.72	−0.50	−0.13	0.00
Spherical Equivalent (D)					
6–8 years	0.59	1.05	0.00	0.50	1.00
9–11 years	0.40	1.33	0.00	0.50	0.75
12–14 years	0.05	1.38	−0.38	0.25	0.54
15–17 years	−0.40	1.77	−0.75	0.00	0.50

## Data Availability

Data is contained within the article or supplementary materials. The original contributions presented in this study are included in the article/Supplementary Material. Further inquiries can be directed to the corresponding author.

## Supplementary Materials

The following supporting information can be downloaded at: https://www.mdpi.com/article/10.3390/jcm14207262/s1, Table S1. Descriptive statistics of sphere, cylinder (negative-cylinder notation), and spherical equivalent (right eye) by individual age and sex.
