# Supplementary material for: Spherical Equivalent Percentile Curves in a Portuguese School-Aged Population
Source: J Clin Med. 2025 Oct 14;14(20):7262. doi: 10.3390/jcm14207262 (PMC12565688; doi:10.3390/jcm14207262)
Supplement: Supplementary file 1 [file jcm-14-07262-s001.zip › jcm-3895809-supplementary.pdf]

Table S1. Descriptive statistics of sphere, cylinder (negative-cylinder notation), and spherical equivalent (right eye) by individual age and sex.

	**Overall (n = 2205)**					**Boys (n = 1087; 49.3%)**					**Girls (n = 1118; 50.7%)**				
	**Mean**	**SD**	**p25**	**p50**	**p75**	**Mean**	**SD**	**p25**	**p50**	**p75**	**Mean**	**SD**	**p25**	**p50**	**p75**
**Sphere**															
**6 years**	0.61	0.96	0.00	0.50	1.00	0.67	0.10	0.00	0.75	1.00	0.57	0.99	0.00	0.50	1.00
**7 years**	0.74	1.20	0.00	0.50	1.00	0.63	1.18	0.00	0.50	1.00	0.83	1.20	0.00	0.63	1.25
**8 years**	0.81	1.26	0.00	0.50	1.00	0.96	1.41	0.25	0.75	1.50	0.63	1.05	0.00	0.50	0.75
**9 years**	0.75	1.67	0.00	0.50	1.00	0.61	1.35	0.25	0.63	1.00	0.85	1.88	0.00	0.50	1.00
**10 years**	0.62	1.31	0.25	0.50	1.00	0.76	1.19	0.50	0.50	1.00	0.46	1.41	0.00	0.50	1.00
**11 years**	0.73	1.43	0.50	0.50	0.75	0.63	1.22	0.50	0.50	0.75	0.84	1.65	0.25	0.50	0.75
**12 years**	0.44	1.00	0.25	0.50	1.00	0.34	1.04	0.00	0.50	1.00	0.61	0.26	0.25	0.50	1.25
**13 years**	0.43	1.44	0.00	0.50	0.75	0.53	1.50	0.00	0.50	0.75	0.33	1.40	-0.50	0.25	0.50
**14 years**	-0.09	1.48	-0.50	0.38	0.60	-0.05	1.14	-0.50	0.25	0.75	-0.13	1.74	-0.50	0.50	0.50
**15 years**	-0.46	2.04	-0.10	0.00	0.50	-0.30	2.43	-0.50	0.00	0.63	-0.62	1.64	-1.25	0.00	0.50
**16 years**	-0.01	1.63	-0.50	0.13	0.50	0.31	1.05	-0.13	0.50	0.50	-0.23	1.91	-0.50	0.00	0.50
**17 years**	-0.04	1.49	-0.25	0.00	0.50	-0.15	1.30	0.00	0.00	0.50	0.05	1.67	-0.25	0.13	0.63
**Cylinder**															
**6 years**	-0.24	0.67	0.00	0.00	0.00	-0.23	0.68	0.00	0.00	0.00	-0.24	0.68	0.00	0.00	0.00
**7 years**	-0.31	0.75	0.00	0.00	0.00	-0.41	0.92	-0.50	0.00	0.00	-0.23	0.57	0.00	0.00	0.00
**8 years**	-0.34	0.87	-0.38	0.00	0.00	-0.44	0.88	-0.50	0.00	0.00	-0.23	0.85	0.00	0.00	0.00
**9 years**	-0.49	0.91	-0.50	0.00	0.00	-0.45	0.96	-0.38	0.00	0.00	-0.52	0.87	-0.50	0.00	0.00
**10 years**	-0.45	0.73	-0.50	-0.25	0.00	-0.39	0.67	-0.50	-0.25	0.00	-0.52	0.80	-0.75	-0.25	0.00
**11 years**	-0.49	0.73	-0.50	-0.25	0.00	-0.42	0.65	-0.50	-0.25	0.00	-0.58	0.81	-0.75	-0.50	0.00
**12 years**	-0.41	0.72	-0.50	0.00	0.00	0.40	0.56	-0.50	-0.25	0.00	-0.41	0.94	-0.25	0.00	0.00

**13 years**	-0.58	0.84	-0.75	-0.50	0.00	0.68	0.94	-0.75	-0.50	0.00	-0.48	0.73	-0.50	-0.25	0.00
**14 years**	-0.47	0.70	-0.50	-0.25	0.00	-0.53	0.77	-0.50	-0.25	0.00	-0.42	0.65	-0.50	-0.25	0.00
**15 years**	-0.62	0.95	-1.00	-0.25	0.00	-0.72	1.05	-1.25	-0.25	0.00	-0.52	0.85	-0.50	-0.25	0.00
**16 years**	-0.40	0.60	-0.50	-0.25	0.00	-0.53	0.76	-0.63	-0.38	0.00	-0.31	0.48	-0.50	-0.13	0.00
**17 years**	-0.31	0.47	-0.50	0.00	0.00	-0.37	0.54	-0.75	0.00	0.00	-0.27	0.41	-0.50	0.00	0.00
**Spherical Equivalent**															
**6 years**	0.58	1.07	0.00	0.50	1.00	0.60	1.16	0.00	0.50	1.00	0.57	0.98	0.00	0.50	1.00
**7 years**	0.59	1.04	0.00	0.50	1.00	0.47	0.99	0.00	0.50	0.81	0.69	1.08	0.13	0.50	1.00
**8 years**	0.59	1.03	0.00	0.50	1.00	0.64	1.05	0.00	0.50	1.00	0.54	1.01	0.00	0.50	1.00
**9 years**	0.40	1.39	0.00	0.50	0.75	0.37	1.18	0.00	0.50	0.88	0.44	1.56	0.00	0.50	0.75
**10 years**	0.37	1.30	0.00	0.50	0.75	0.43	1.17	0.00	0.50	0.75	0.30	1.43	0.00	0.50	0.75
**11 years**	0.44	1.28	0.00	0.50	0.75	0.36	1.05	0.00	0.50	0.75	0.53	1.49	0.00	0.50	0.75
**12 years**	0.21	1.09	0.00	0.38	0.75	0.22	1.15	0.00	0.50	0.75	0.20	1.00	-0.13	0.25	0.75
**13 years**	0.14	1.41	-0.44	0.25	0.50	0.19	1.55	-0.38	0.25	0.50	0.09	1.27	-0.50	0.13	0.50
**14 years**	-0.32	1.70	-0.63	0.25	0.50	-0.31	1.30	-0.50	0.19	0.50	-0.33	2.00	-0.75	0.31	0.50
**15 years**	-0.76	2.11	-1.50	0.00	0.38	-0.66	2.62	-1.25	0.00	0.50	-0.84	1.57	-1.50	-0.13	0.25
**16 years**	-0.21	1.61	-0.75	0.06	0.50	0.05	1.20	-0.25	0.25	0.50	-0.39	1.82	-0.88	0.00	0.38
**17 years**	-0.20	1.42	-0.38	0.00	0.25	-0.34	1.26	0.00	0.00	0.25	-0.08	1.57	-0.50	0.06	0.50

The measurements of sphere, cylinder (negative-cylinder notation) and spherical equivalent were taken in the right eye.
